# Supplementary material for: Yiai Fuzheng decoction inhibits triple-negative breast cancer by remodeling the immune microenvironment
Source: Front Immunol. 2025 Sep 30;16:1615631. doi: 10.3389/fimmu.2025.1615631 (PMC12518410; doi:10.3389/fimmu.2025.1615631)
Supplement: Supplementary file 2 [file DataSheet1.zip › Supplementary File 2/Treatment/C_vs_H/07_Pathway_Analysis/Pathway_Analysis/Pathway_Barplot.pdf]

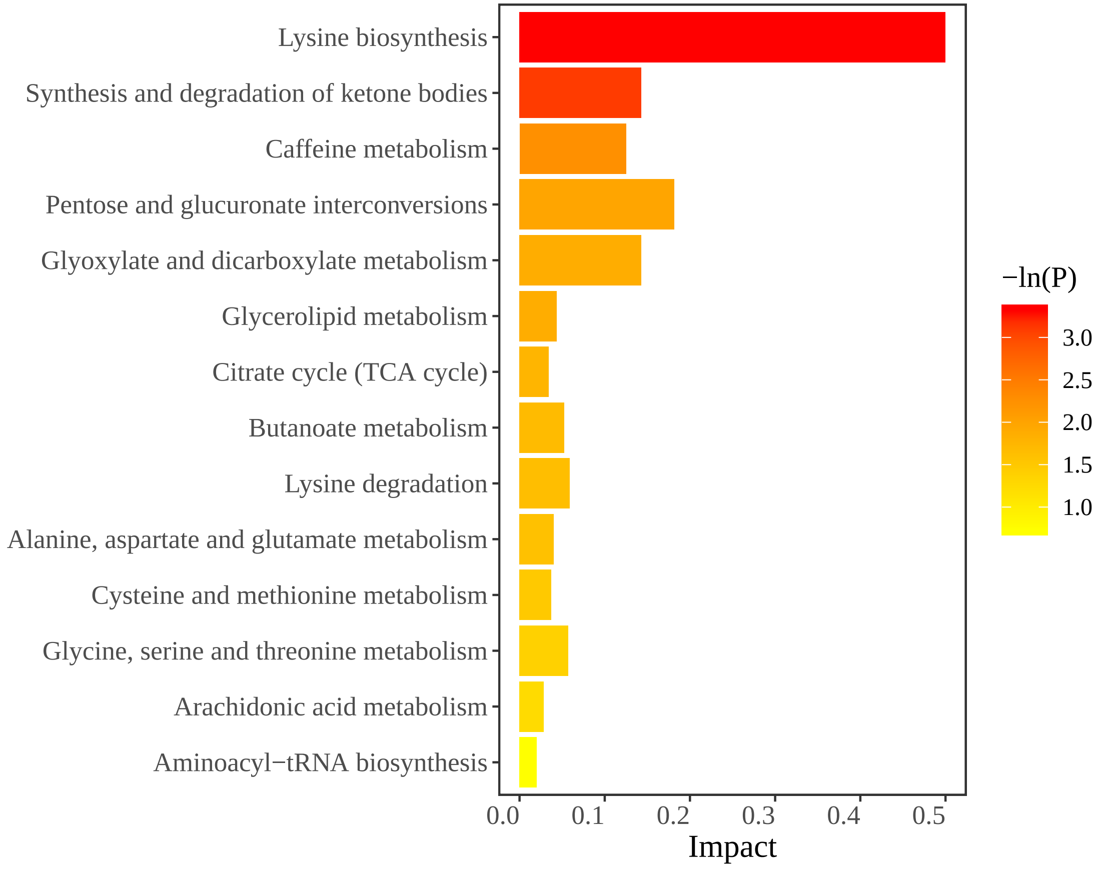
Lysine biosynthesis
Synthesis and degradation of ketone bodies
Caffeine metabolism
Pentose and glucuronate interconversions
Glyoxylate and dicarboxylate metabolism
Glycerolipid metabolism
Citrate cycle (TCA cycle)
Butanoate metabolism
Lysine degradation
Alanine, aspartate and glutamate metabolism
Cysteine and methionine metabolism
Glycine, serine and threonine metabolism
Arachidonic acid metabolism
Aminoacyl−tRNA biosynthesis
0.0
0.1
0.2
0.3
0.4
0.5
Impact
−ln(P)
3.0
2.5
2.0
1.5
1.0
